# Genetic newborn screening and digital technologies: A project protocol based on a dual approach to shorten the rare diseases diagnostic path in Europe

**DOI:** 10.1371/journal.pone.0293503

**Published:** 2023-11-22

**Authors:** Nicolas Garnier, Joanne Berghout, Aldona Zygmunt, Deependra Singh, Kui A. Huang, Waltraud Kantz, Carl Rudolf Blankart, Sandra Gillner, Jiawei Zhao, Richard Roettger, Christina Saier, Jan Kirschner, Joern Schenk, Leon Atkins, Nuala Ryan, Kaja Zarakowska, Jana Zschüntzsch, Michela Zuccolo, Matthias Müllenborn, Yuen-Sum Man, Liz Goodman, Marie Trad, Anne Sophie Chalandon, Stefaan Sansen, Maria Martinez-Fresno, Shirlene Badger, Rudolf Walther van Olden, Robert Rothmann, Patrick Lehner, Christof Tschohl, Ludovic Baillon, Gulcin Gumus, Edith Gross, Rumen Stefanov, Georgi Iskrov, Ralitsa Raycheva, Kostadin Kostadinov, Elena Mitova, Moshe Einhorn, Yaron Einhorn, Josef Schepers, Miriam Hübner, Frauke Alves, Rowan Iskandar, Rudolf Mayer, Alessandra Renieri, Aneta Piperkova, Ivo Gut, Sergi Beltran, Mads Emil Matthiesen, Marion Poetz, Mats Hansson, Regina Trollmann, Emanuele Agolini, Silvia Ottombrino, Antonio Novelli, Enrico Bertini, Rita Selvatici, Marianna Farnè, Fernanda Fortunato, Alessandra Ferlini

**Affiliations:** 1 Pfizer Inc., Collegeville, Pennsylvania, United States of America; 2 KPM Center for Public Management and Swiss Institute for Translational and Entrepreneurial Medicine, University of Bern, Bern, Switzerland; 3 Department of Mathematics and Computer Science, University of Southern Denmark, Odense, Denmark; 4 Department of Neuropediatric and Muscle Disorders, Medical Center, Faculty of Medicine, University of Freiburg, Freiburg, Germany; 5 Takeda Pharmaceuticals International AG, Opfikon, Switzerland; 6 Department of Neurology, University Medical Center Goettingen, Göttingen, Germany; 7 F. Hoffmann La-Roche, Basel, Switzerland; 8 Novo Nordisk Health Care AG, Switzerland &Novo Nordisk A/S, Kloten, Denmark; 9 University College Dublin, National University of Ireland, Dublin, Ireland; 10 Lysogene, Neuilly-sur-Seine, France; 11 Sanofi, Diegem, Belgium; 12 Illumina, Cambridge, United Kingdom; 13 Novartis Gene Therapies Switzerland GmbH, Risch-Rotkreuz, Switzerland; 14 Research Institute AG & Co KG, Digital Human Rights Center, Wien, Austria; 15 PTC Therapeutics Switzerland GmbH, Steinhausen, Switzerland; 16 EURORDIS, Paris, France; 17 Department of Social Medicine and Public Health, Faculty of Public Health, Medical University of Plovdiv, Plovdiv, Bulgaria; 18 Bulgarian Association for Promotion of Education and Science, Institute for Rare Disease, Plovdiv, Bulgaria; 19 Genoox, Tel Aviv, Israel; 20 Berlin Institute of Health (at) Charité - Universitätsmedizin Berlin, Berlin, Germany; 21 Translational Molecular Imaging, Max Planck Institute for Multidisciplinary Sciences, Göttingen, Germany; 22 Clinic of Hematology and Medical Oncology, University Medical Center, Göttingen, Germany; 23 Institute for Diagnostic and Interventional Radiology, University Medical Center, Göttingen, Germany; 24 Swiss Institute for Translational and Entrepreneurial Medicine (sitem-insel), Bern, Switzerland; 25 SBA Research gGmbH, Wien, Austria; 26 Medical Genetics, University of Siena, Siena, Italy; 27 Bulgarian Association for Personalized Medicine, Sofia, Bulgaria; 28 Centro Nacional de Analisis Genomico, CNAG, Barcelona, Spain; 29 FindZebra APS, Copenhagen, Denmark; 30 Department of Strategy and Innovation, Copenhagen Business School, Copenhagen, Denmark; 31 Uppsala University, Uppsala, Sweden; 32 University of Erlangen, Erlangen, Germany; 33 Bambino Gesu’ Children Hospital, IRCCS, Rome, Italy; 34 Medical Genetics, Department of Medical Sciences, University of Ferrara, Ferrara, Italy; PLOS: Public Library of Science, UNITED KINGDOM

## Abstract

Since 72% of rare diseases are genetic in origin and mostly paediatrics, genetic newborn screening represents a diagnostic “window of opportunity”. Therefore, many gNBS initiatives started in different European countries. Screen4Care is a research project, which resulted of a joint effort between the European Union Commission and the European Federation of Pharmaceutical Industries and Associations. It focuses on genetic newborn screening and artificial intelligence-based tools which will be applied to a large European population of about 25.000 infants. The neonatal screening strategy will be based on targeted sequencing, while whole genome sequencing will be offered to all enrolled infants who may show early symptoms but have resulted negative at the targeted sequencing-based newborn screening. We will leverage artificial intelligence-based algorithms to identify patients using Electronic Health Records (*EHR*) and to build a repository “*symptom checkers*” for patients and healthcare providers. S4C will design an equitable, ethical, and sustainable framework for genetic newborn screening and new digital tools, corroborated by a large workout where legal, ethical, and social complexities will be addressed with the intent of making the framework highly and flexibly translatable into the diverse European health systems.

## Background

### Rare disease state-of-the-art and impact on health system(s)

Achieving early diagnosis and delivering effective treatments to people living with rare disease (PLWRD) have been described as two of the major global health challenges for the 21st century. There are approximately 6000 to 7000 highly heterogenous rare diseases (RDs) resulting in 30 million PLWRD in Europe and 250 to 450 million globally [1–3]. About 75% of RDs are genetic in origin, which implies that a genetic diagnosis is compulsory in order to achieve a complete diagnosis. Although the majority of genetic RDs are monogenic, several RD-causing risk variants are spread across the genome with complex inheritance patterns. Less than 5% of RD have an existing treatment [3, 4].

RDs span across a plethora multisystemic syndromes, involving virtually every single organ or physiological function, therefore they heavily engage health systems (HSs) resources and professionals. Conversely, most PLWRDs have common needs, as reported by Eurordis [5] which include:

- Lack of access to appropriate diagnostic approaches;
- Diagnostic odyssey (prolonged time to diagnosis);
- Insufficient scientific knowledge on the disease, including clinical presentation and natural history;
- Heavy social and psychological consequences of diagnosis;
- Lack of appropriate quality healthcare once diagnosis is reached;
- Inequities across all these aspects

These hurdles do cause an heavy demand of care to HSs, which might be however ineffective in meeting the patients’ needs since of the lack of diagnosis.

### Rare disease diagnosis and impact on quality of life

The not-so-rare “Diagnostic Odyssey”: many RDs are chronic and often progressive, and the clinical worsening could be delayed or better managed with appropriate treatment/care where treatment or active clinical research exists. Therefore, early diagnosis is key, not only to the psychosocial well-being of the individual and their caregivers, but also to their health. The challenges stem from multiple reasons. First, physicians are trained to recognise “horses”, not “zebras”, meaning that the medical community is trained to look first for the more common diseases. Further complicating the matter, RDs are characterized by a broad diversity of disorders and symptoms that vary not only from disease to disease, but also from patient-to-patient suffering from the same disease. The symptoms themselves can often also reflect more common conditions, thus, leading to a misdiagnosis, or a lengthy and inefficient rule-out process. Altogether, this leads to a long and burdensome path to diagnosis that can take 8 years for an average case [6] often superposed with misdiagnosis, unnecessary, sometimes harmful diagnostic procedures and pointless treatments. This creates a heavy human [7] and societal cost, estimated at 1 trillion dollars in the US in 2019 [8] that could be mitigated by earlier diagnosis.

### The rare disease conundrum: A cascade of effects of being “undiagnosed”

Despite the recent rise in rare disease research and development, the vast majority of RDs remain under-studied, and therefore under-treated/-cared for. This can be attributed for the most part to the fact that PLWRD are often not diagnosed, which leads to a lack of epidemiological and natural history data. In turn this translates into a lack of validated endpoints, and few patient-reported outcomes instruments or clinical severity scales. These hurdles tend to stand in the way of Research & Development (R&D), compounded by the fact that PLWRD are few by definition (thus–a limited pool of potential research volunteers or clinical trial participants is available); experts for a particular RD are even fewer and invariably belonging to excellence centers, thus a limited pool of potential investigators, difficult to reach for patients. This has the negative effect of blunting diagnosis and/or newborn screening initiatives at the policy level, as it would lead to PLWRD being diagnosed, with no approved treatment available (see Table 1, left), because most health systems in Europe utilize the Wilson and Jungner’s newborn screening criteria in 1968 [9], which was commissioned by the World Health Organization and is frequently referred to as the “gold standard” for selecting diseases to be screened [10].

**Table 1:** The Wilson and Jungner’s principles for the early detection of disease and the 5 Recommendations for RDs.

The principles proposed by Wilson and Jungner (1968) for the early detection of disease		Matching content of the 5 Recommendations
1	The condition sought should be an important health problem	Recommendation 1
2	There should be an accepted treatment for patients with recognized disease	Recommendation 5
3	Facilities for diagnosis and treatment should be available	Recommendation 2,3
4	There should be a recognizable latent or early symptomatic stage	Recommendation 1
5	There should be a suitable test or examination	Recommendation 2
6	The test should be acceptable to the population	Recommendation 2
7	The natural history of the condition, including development from latent to declared disease, should be adequately understood	Recommendation 2, 5
8	There should be an agreed policy on whom to treat as patients	Recommendation 2, 5
9	The cost of case-finding (including diagnosis and treatment of patients diagnosed) should be economically balanced in relation to possible expenditure on medical care as a whole	Recommendation 1, 3, 5
10	Case-finding should be a continuing process and not a “once and for all” project	Recommendation 1, 3, 5

In light of the enormous amount of knowledge about RDs today available, thanks to the genetic revolution and the identification of new therapies, the 10 criteria might deserve to be revised and updated. Indeed, a joint international initiative, facilitated by EURORDIS and co-signed by patient advocacy organisations from the UK, Australia, Japan, and the US, has delivered 5 Recommendations to address specific needs of undiagnosed rare disease patients [11]. If we try to “adapt” the Wilson and Jungner’s criteria into these recent 5 Recommendations, we may understand whether and to what extent the patients’ view were recapitulated and may have played a role into the original NBS criteria formulation (see Table 1, right).

**Fig 1:**
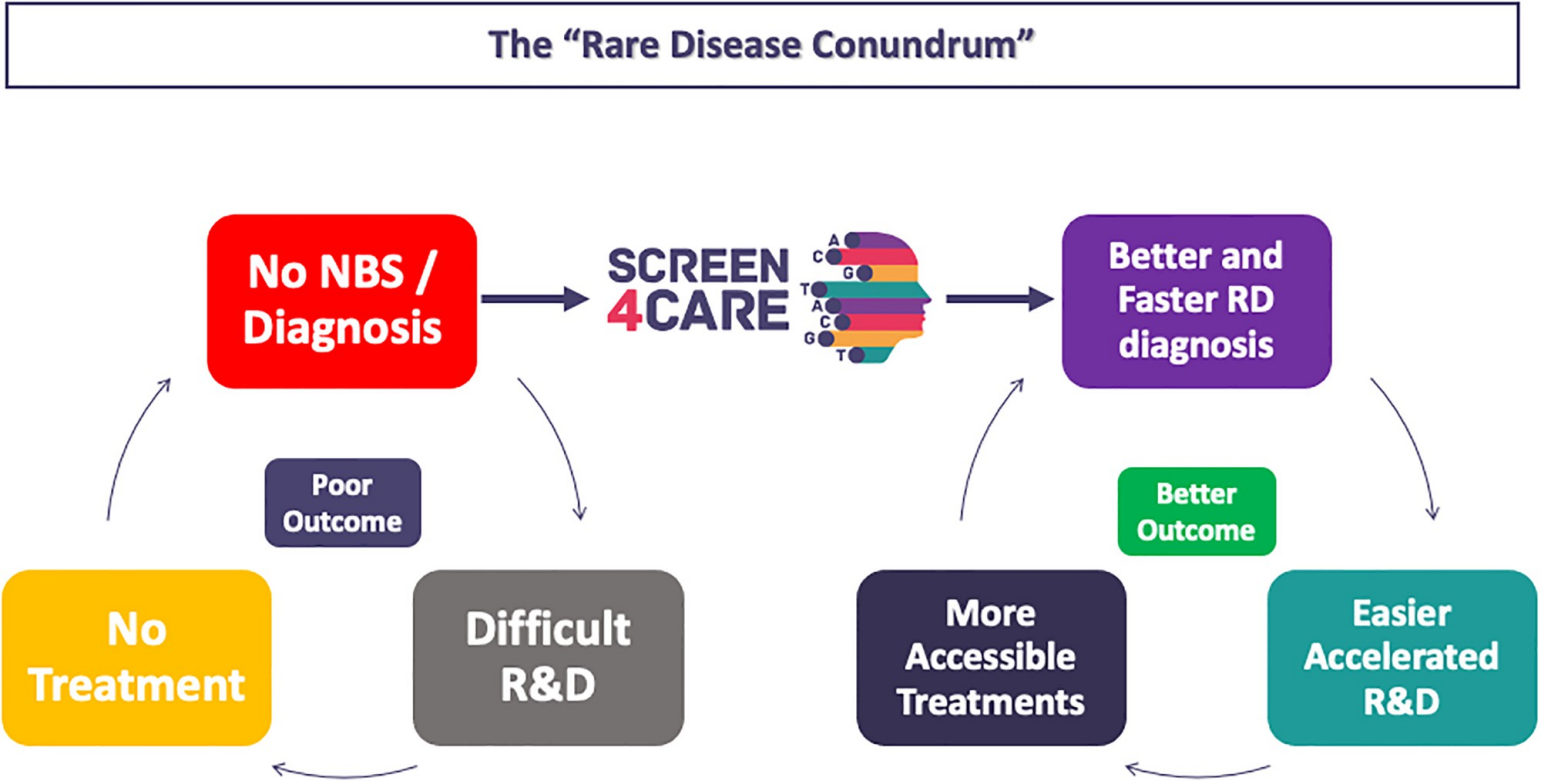
Representation of main factors contributing to the vicious circle (the “rare disease conundrum”) that prevents the acceleration of rare disease clinical trials, overall R&D, and better outcome for PLWRD. Strategies implemented by S4C to break this loop are also represented in the figure. Abbreviations: NBS, Newborn screening; RD, Rare Diseases; R&D, Research and Development.

Interestingly, but not surprisingly, Recommendation 4 (Patients should be equally involved.) does not seem to contribute to the Wilson and Jungner’s criteria formulation. Indeed, only recently, PLWRD are considered as main actors and co-creators of the health care path design. In particular, criteria 2, 4 and 7 may deserve further discussion, since it might be difficult if not impossible they can be met properly in ultrarare RDs. The RD and NBS communities should therefore promote a forum with all actors involved to discuss about novel strategies and future policies of NBS. Fig 1 summarises the RD “conundrum” and cascade of effects.

## Rationale

### Genetic newborn screening: The “zero point in time” of RDs’ diagnosis

Genetic newborn screening (gNBS) represents an innovative, accurate, and scalable method to accelerate RDs diagnosis. As nearly 50 to 75 percent of RDs begin in childhood [12], a gNBS program may greatly impact on early diagnosis in children. Since the Human Genome Project, the cost of DNA sequencing has progressively decreased, to the extent that from 2020ies gene sequencing by high throughput technologies, applied with high scalability, has become more accessible for screening procedures. With advances in innovative sequencing approaches [13], combined with our increased capacity to collect, store, process and interpret massive amounts of data (“big data”), we have reached a collective, unprecedented milestone in healthcare history providing the opportunity to significantly transform the landscape of RDs diagnosis as it is today.

## The Screen4Care EU-IMI project

In 2021 the EU-IMI initiative launched the public-private-patient consortium Screen4Care (S4C) which aims to tackle this challenge head on by focusing on shortening the path to diagnosis for PLWRD, from three angles: i) mapping of existing converging resources and initiatives, ii) designing and running a genetic newborn screening, iii) applying artificial intelligence to phenotypic recognition.

S4C is therefore committed to accelerate the diagnosis of RD, which will positively impact PLWRD quality of life and RD research.

**Fig 2:**
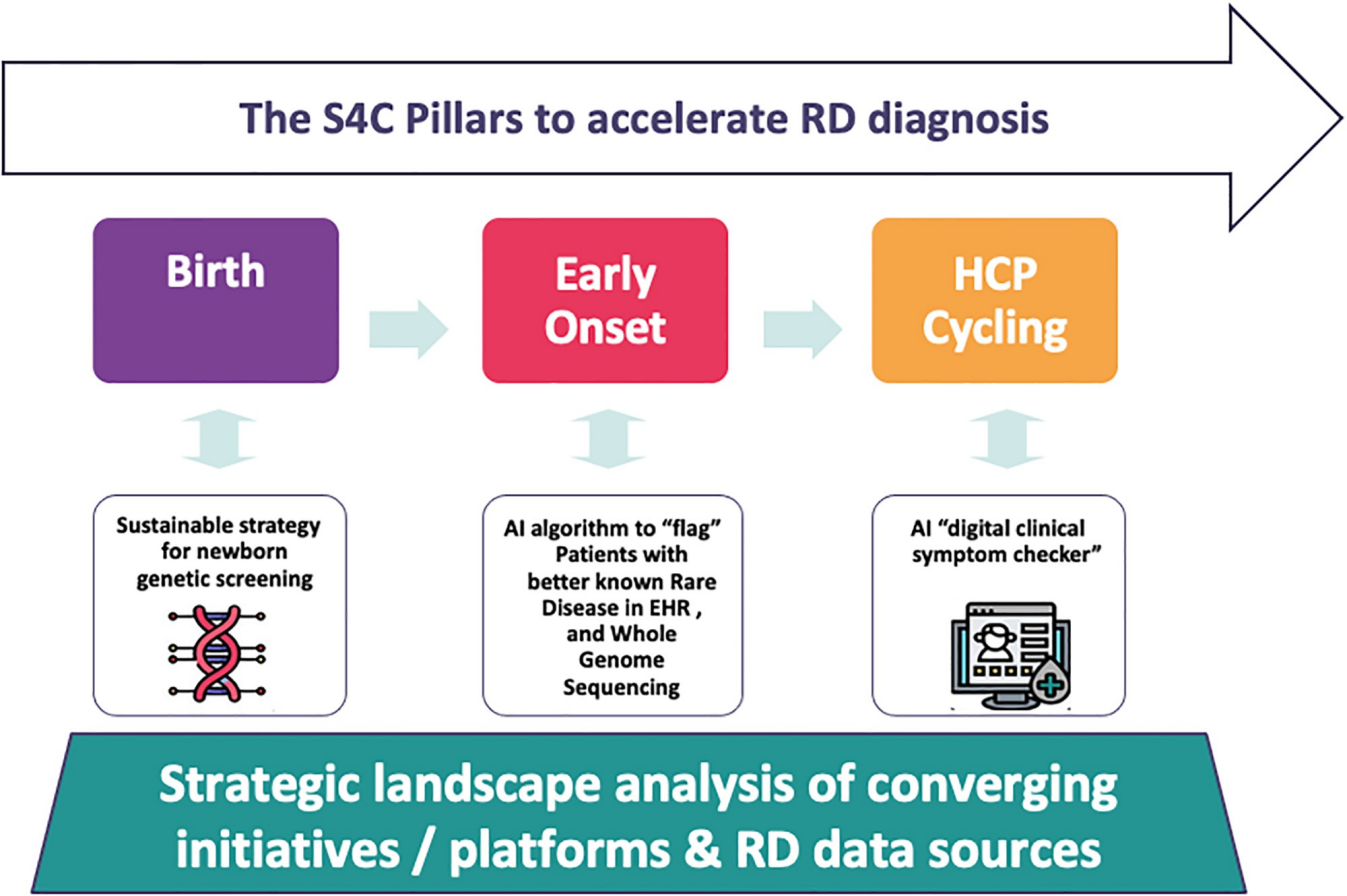
The S4C pillars with multi-pronged dual strategy to shorten RD diagnosis. Abbreviations: NBS newborn screening, HCP health care provider, EHR, Electronic Health Records, RD research & development.

Screen4Care is an international public-private-patient-partnership (PPPP) consortium, launched on October 1^st^, 2021, and intended to run for a period of five years with a total budget of EUR 25 million provided by the Innovative Medicines Initiative (IMI 2 JU), a joint undertaking of the European Union and the European Federation of Pharmaceutical Industries and Associations (EFPIA). The Screen4Care team brings together 20 academic partners, 11 industrial project partners, and 4 small and medium-sized enterprises. Furthermore, the consortium is enriched by the active participation of EURORDIS, representing the voice of PLWRD in Europe to promote a robust and meaningful dialogue as well as to ensure that the needs and preferences of the rare disease patient community guide the progression of the project. The S4C research consortium is co-led by Italy, University of Ferrara (scientific coordinator) and by Pfizer Inc (project leader). A scheme illustrating the S4C main pillars in described in Fig 2.

Strategic collaboration between Private and Public Partners is paramount to this project, as it is positioned at the cusp between Research and Public Health Policy and results will lead the S4C consortium to propose experimentally tested, detailed, and disease-oriented approaches which will serve to develop/put forward recommendations for policy makers and national health systems. Perspectives from Public Partners will ensure that proposed solutions are fit-for-purpose, and that they truly add value for all stakeholders. A PPPP offers a unique framework for all parties to engage in delivering the range of input and expertise necessary for achieving the project aims and ensuring that a practical and long-term sustainable plan will be actioned.

### Scree4care and its impact on RD community

**Fig 3:**
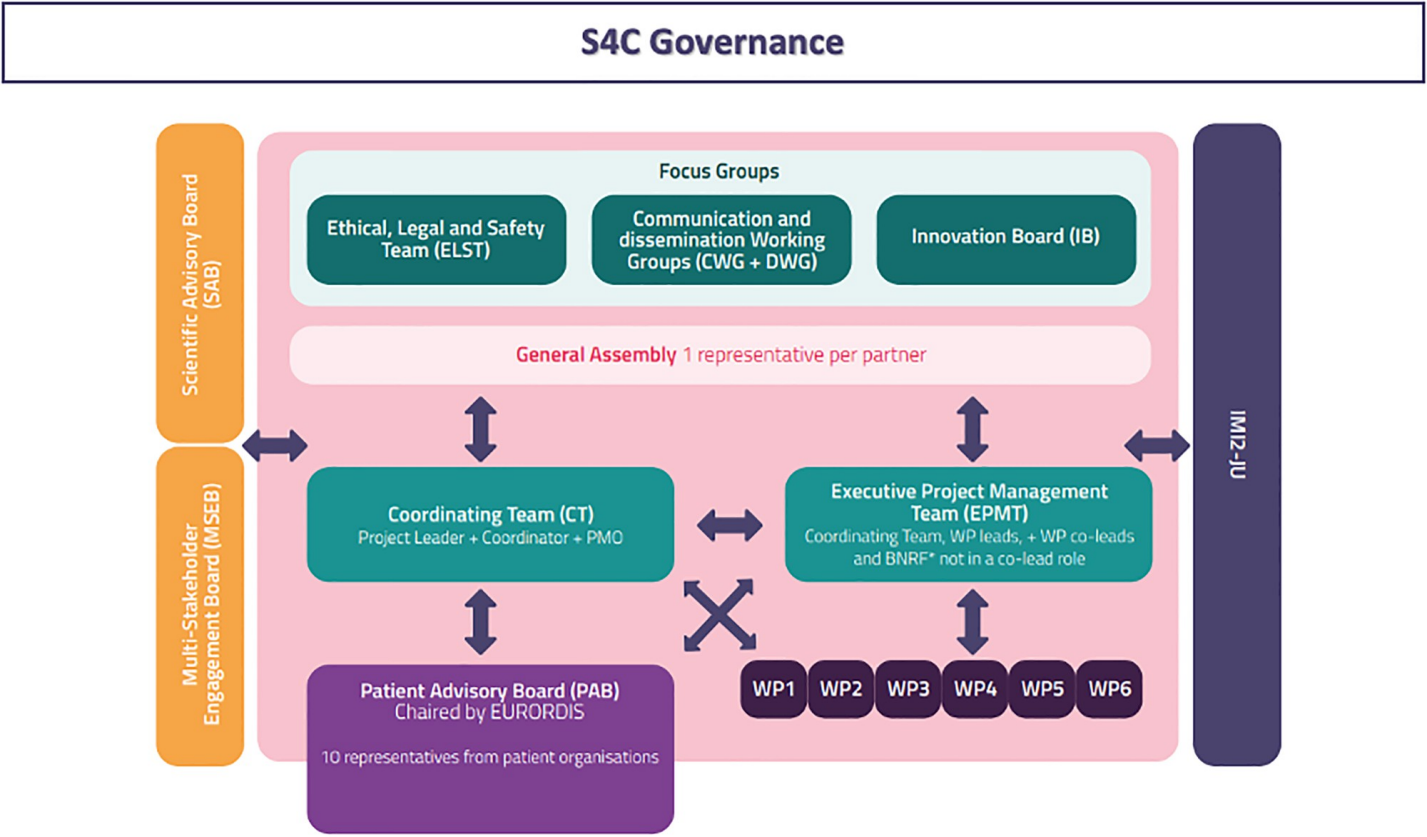
Schematic representation of the multi-layered structure of S4C and its governance and board composition to ensure appropriate levels of cooperation, scientific exchange, multi-stakeholder engagement, dialogue, and cooperation with other initiatives (as IMI2-JU). Abbreviations: ELST, Ethical, Legal and Safety Team; CWG, Communication Working Group; DWG, Dissemination Working Group; IB, Innovative Board; CT, Coordinating Team; PMO, Project Management Office; EPMT, Executive Project Management Team; WP, Work Packages; PAB, Patient Advisory Board; SAB, Scientific Advisory Board; MSEB, Multi-Stakeholder Engagement Board; IMI2-JU, Innovative Medicine Initiative 2 Joint Undertaking, WP workpackage.

S4C has an innovative research approach combining genomic strategies and digital technology. The project consists of 6 workpackages (WPs), 5 of them being research activities, and the last managing activities. The WPs are summarized in Fig 3, where the general S4C structure, including the several dedicated boards, is described. The main impacts of the project are listed in Table 2.

**Table 2:** Expected impact of Screen4Care on patients, research, healthcare, and society.

Expected Impact of Screen4Care	
Benefits to Patients: Decreased time to the right diagnosis Improved patient journey Better healthcare Increased quality of life Decreased irreversible harm Informed reproductive choices	Benefits to Research: Advances in utilization of digital technologies Increased disease knowledge for future research Improved data availability for future research Better RDs epidemiological data Better RDs natural history data More robust R&D in RD
Benefits to Healthcare: Implementation of digital transformation in healthcare Paradigm change in rare diseases diagnosis Improved diagnostic tools Improved understanding of disease Higher accuracy in clinical decisions Better care delivery Integrated care among different specialties	Benefits to Society: Decreased burden for family and caregivers Increased reliance in the healthcare system Better use of data for public health Improved value-based healthcare

## Objectives and methodologies

### Ethical issues

Screen4Care project and related protocol was fully approved by European Commission and Innovative Health Initiative Ethical Committee, the 16^th^ of April 2021, Ref. document Ares (2021) 2586772.

### Scree4care and pillar activities

#### Pillar 1: Strategic landscape analysis: The Screen4Pedia

Pillar 1 (WP1) lays the foundations for understanding the business, legal, ethical, and regulatory environment for RD screening in Europe and accompanies the project with stakeholder engagement activities. We will conduct three landscape analyses. First, we identify ongoing and planned RD screening activities in Europe to be able to liaise with such initiatives and to complement them by adding value whenever possible and relevant. Second, we perform a landscape analysis of available data sources to serve as a starting point for developing machine-learning (ML) algorithms to identify rare conditions in electronic health records (EHRs) (Pillars 2 & 4). Third, we conduct a horizon scan on available symptom checker initiatives that builds the basis for Pillar 5, the development of a meta-symptom checker for rare diseases.

To comply with ethics and legal requirements, we develop a Data Protection Impact Assessment Framework. The application of the framework ensures that the data of individuals is protected from a legal and technical point of view and the framework lays the foundations to ensure comprehensive data safety and security. In addition, we develop a Code of Ethical Practice to serve as guiding document for ethically sharing and accessing personal data throughout the project. Pillar 1 is working closely with the internal Ethics, Legal, and Safety Team (ELST) and the Independent Ethics Advisor in order to address all ethical and legal issues properly. To be mentioned that he ELST and the independent Ethics advisor are cross-cutting through the whole project such as the Patient Advisory Board (PAB), the multistakeholder board (MSEB) or the transversal innovation group.

We assess the regulatory landscape requirements to place advanced AI screening technologies in the market in order to bring new ML-based technologies to healthcare providers and patients in Europe efficiently. Specifically, we develop a guideline for regulatory approval and compliance requirements that need to be addressed by the manufacturers. Furthermore, we analyse questions related to funding, reimbursement, and adoption of medical technologies of genetic and machine-learning based screening technologies that allows for determining sustainable business models for companies and healthcare providers. Pillar 1 is complemented by continuously engagement with stakeholders to discuss project results and disseminate the key messages to PLWRD, international screening societies, and other relevant groups.

#### Pillar 2 (WP2): Federated meta-data repository

Medical data is scattered around the globe in different hospitals, specialized public or private databases and research institutes in so-called data silos. The data are often stored in incompatible formats, meaning that the information of one dataset cannot easily, if at all, be combined with the information of another dataset. This hampers research and the application of modern machine learning tools which require large amount of data to be trained sufficiently; and the problem worsens when considering rare diseases with consequentially low incidence numbers per data silo. This renders the application of machine learning for rare diseases difficult, if no solution to this data sparsity is found. The obvious solution of consolidating the data into a single cloud fails due to regulatory restrictions and privacy considerations. Pillar (Work package) 2 plans to tackle these issues by creating a FAIR (Findable, Accessible, Interoperable, Reusable) research environment. Firstly, to make data findable, we will develop a metadata repository, which does not store the actual data, but information about the data (metadata) which remains in the data silos. Therefore, with the metadata repository, we can identify where data is located, for example which databases contain data of patients with a disease of interest and identify compatible datasets. Secondly, to ensure interoperability and reusability, we are working on a Screen4Care Implementation Guide for Rare Disease Documentation (IGRDD). The S4C IGRDD has two goals: it serves as a guideline for action in the preparation of existing data from care and research for secondary research use, and it serves as a guideline for the design of new RD documentation in clinical department information systems, RD electronic data capture systems and RD registers. This approach will serve to analyse and use common data elements of the European Rare Disease Registry Infrastructure, as those carried on by European Reference networks (ERNs), therefore building upon existing standards to ensure maximal compatibility with existing data sources. When all relevant and compatible data sources are identified, to ensure that they can be accessible to train and test artificial intelligence (AI) algorithms, we will develop new algorithms, tailored for low incidence classes in an innovative federated fashion: the data remains at the data silos, but the machine learning comes to the data. These local machine models will help to identify undiagnosed RD patients, whom phenotype is represented in the datasets. Importantly for the legal and ethical aspects, at no point do the sensitive medical data ever leave the site of storage, and continuous data protection assessment will be carried out. A consultative co-design process will be adopted by involving user different groups to addressing the needs of RDs patients and it supports efforts across the entire project, especially pillar 4 and 4, see below, by including regular involvement of co-creation participants at each stage of project research, development and testing.

#### Pillar 3 (WP3): Genetic newborn screening for RDs and early diagnosis in young symptomatic infants

Pillar 3 aims at exploring the use of gNBS in newborn as gateway to shorten the diagnostic path and offer the opportunity for early treatment. This pillar is composed of different tasks. Preference studies will be carried out on expecting couples, PLWRD and caregivers, healthcare professionals and the general public, to provide input to the gNBS design, data flow, and compliance. Insights resulting from these stakeholder preference studies will help to propose a gNBS framework which might be acceptable, ethical, and sustainable, therefore encountering large consensus among all stakeholders.

**Fig 4:**
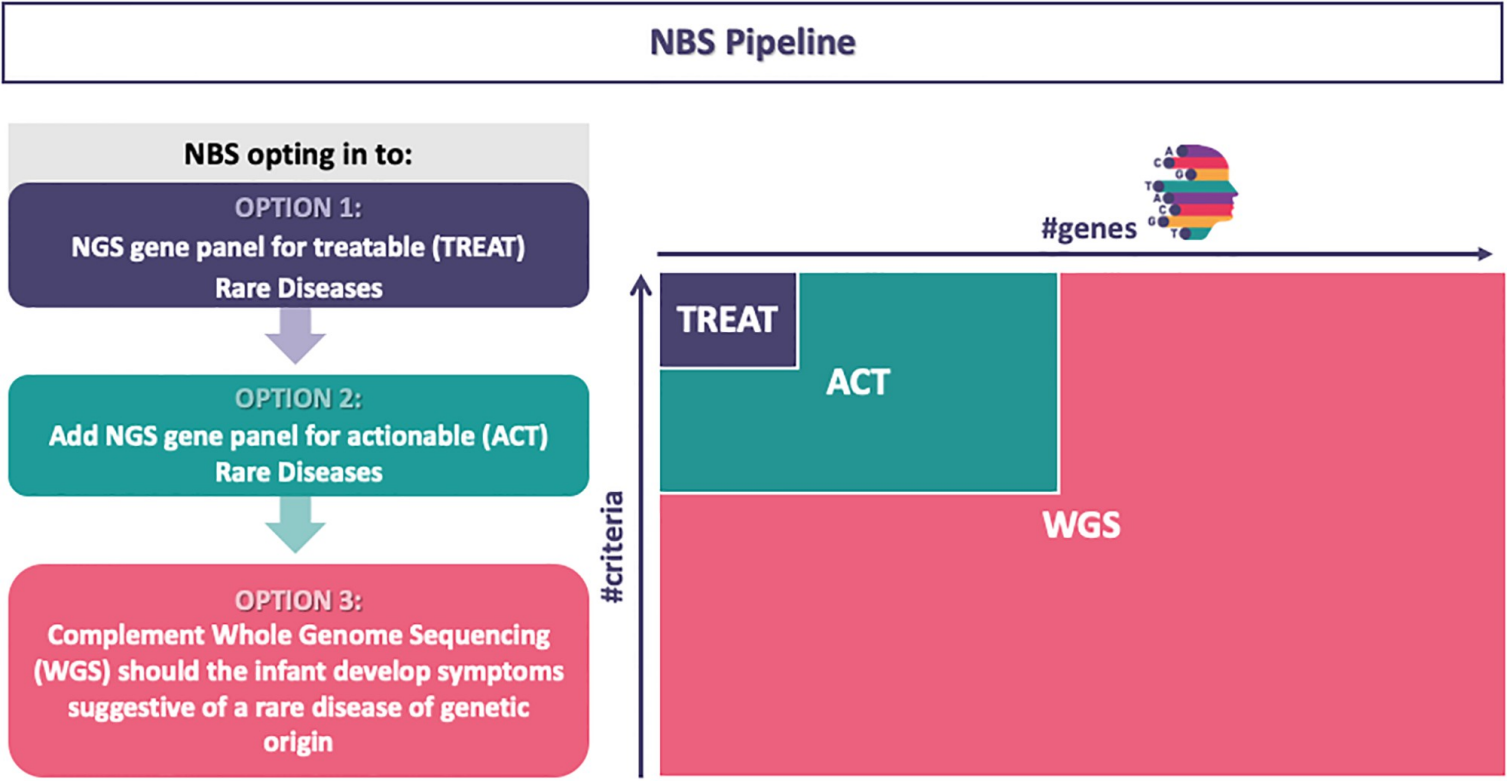
Different strategies adopted to drive gNBS. Option 1) Currently treatable RDs (TREAT-panel-approach); Option 2) Actionable RDs (ACT-panel-approach); Option 3) Whole Genome Sequencing (WGS) for early symptomatic infants, tested negatively during panel-based gNBS, to recognize known NBS targeted sequencing based-escaped RDs and/or novel genes/phenotypes. On the right panel, the comparison and proportion of the final number (#) of genes screened using the three different approaches is shown. Robust criteria will be applied to select and prioritize genes to be included in TREAT and/or ACT panels. Abbreviations: NGS next generation sequencing, NBS newborn screening.

gNBS will be carried out using gene panels which design will include treatable (TREAT) and actionable (ACT) RDs. The gene/disease selection is a crucial step in gNBS planning, therefore robust criteria and scoring will be applied to design the gene panels. These will be used in a large pilot gNBS study that will run in several clinical sites in 4 European countries. Inclusion criteria will be on availability and accessibility of treatment (TREAT panel) and complemented with further criteria on knowledge of the disease, clinical utility and aspects relating to the genetic sequencing technology. The TREAT panel will include about 200 genes that are related to treatable genetic disorders, prioritizing those with early onset and where natural history key elements are known. Secondly, informed by literature research, extensive consulting with various experts and stakeholders, and input that will be gained from surveys via the EURORDIS Rare Barometer, an ACT panel will be designed to include a wider set of disease beyond treatable conditions, that are considered “actionable” [5]. The TREAT (and ACT) arm of the pilot gNBS is targeting to enrol at least 18.000 and up to 30,000 (thus about 25.000) newborn in EU. The choice of using targeted sequencing and gene panel maximizes the screening ability to enrich for interpretable variation, including copy number variations and complex genotypes, provides a more understandable expectation to parents, allows rapid data processing and cost-effective data storage, and avoids many of the complex ethical issues associated with incidental findings and participant privacy and legal aspects, inherent in whole exome or whole genome sequencing, being fully adherent to the World Health Organization ethical guidelines for genetic testing (https://apps.who.int/iris/handle/10665/63910, 1997). However, when infants present with early symptomatic disease, those who tested negative on the TREAT (and/or ACT) panel will be offered optional Whole Genome Sequencing (WGS) to recognize known gNBS-escaped RDs and novel genes/phenotypes. In addition, communications with ERNs (https://webgate.ec.europa.eu/ern/) will help map out post genetic report diagnosis work up and referrals, and correct therapies addressing. Pillar 3 will also collect some data on stakeholder preference and economic impact with the objective to help shape an ethical and sustainable gNBS ecosystem in Europe. Genetic strategies are described in Fig 4.

#### Pillar 4 (WP4): Harnessing artificial intelligence to leverage electronic health records

WP4 will develop AI-based algorithms to scan electronic health records (EHR) and generate “symptom checker apps”, which we intend to make available as tools for clinicians, genetic counsellors, patients, and the general public, to support patients in the early stages of disease by enabling faster diagnosis, supporting symptom-based diagnosis at any age, also beyond the neonatal period. Metadata repository will be used to identify cohorts, which are scattered around several data silos and form the basis for the training of the artificial intelligence algorithms. The trained ML models will be carefully evaluated and tested for their practical applicability. Pilot studies, including analysis of effects of EHR-based diagnostics on pilot participants, as well as a cost-effectiveness analysis, will enable to recognize and highlight ethical, legal, socio-economic and safety aspects that may become relevant to users especially patients, healthcare providers and society.

Another part of Pillar 4 is dedicated to the phenotypic characterisation of neuromuscular diseases, with the aim of identifying new biomarkers based on imaging parameters, such as high-resolution multiphoton imaging and multispectral optoacoustic tomography.

#### Pillar 5 (WP5): Symptom checkers and virtual clinics

The goal of WP5 is to design the Screen4Care Platform which includes Meta-Symptom Checker and Virtual Clinics, to help PLWRD who are “cycling” with their symptoms and moving from one healthcare provider or specialist to another in search of diagnosis or ‘answers’. PLWRD often search the internet and use “unvalidated” online symptom checkers to understand the cause of their symptoms.

With the S4C meta-symptom checker we will begin with reference to the horizon scan, updated regularly throughout the project to ensure timely coverage of state of the art. Based on that scan, and informed by our ongoing inclusive co-design methodology in the co-creation process of the project overall, we aim to connect from our novel open source platform to existing symptom checkers, and to curate the recommendations, presenting them in a user-friendly and meaningful way for PLWRD and their families, as well as for clinicians. The Meta-Symptom Checker is not meant to replace appropriate diagnosis by trained medical professionals, but rather to provide a clinical decision support tool that will reduce the risk of misdiagnosis and lead times to the most appropriate diagnosis and medical management. The meta-symptom checker will be embedded in the S4C virtual platform which will also host a “virtual clinic” room with specific safe online spaces for users to engage in specific types of chat and information/experience exchange with others (whether with other ERNs will be again involved in the project by dedicated initiatives as clinical and diagnostic key players, which already act in a EU structured network offering clinical care to all RD types. An impact analysis will highlight measures of success in terms of patient engagement, patient/family advocacy and return visits to the virtual clinics to continue contact with the larger RDs community beyond diagnosis. Overall, the platform will bring real world supports to patients and their families, and will provide HCPs with a safe, stable, and innovative space for engagement and sharing of expertise in time. This model and its validated outputs may serve as a prototype to design digital tools to be adopted by the health systems.

## Organization of the research project: Governance and boards

A consortium of this size and nature needs a robust structure in order to be managed appropriately and successfully. The Consortium Agreement defines responsibilities and obligations of all partners is in place, binding the actions and commitment of all the partners. Sharing information and updates with the community is also a very important aspect of S4C. This is why we plan to have all S4C publications “open access”. The IMI consortium agreement provides the legal framework for IP-management, and an exploitation strategy is being developed. This is further supported by the S4C Innovation Board. Regulatory compliance is ensured by establishing communication with representatives of relevant regulatory authorities. Early engagement with regulatory bodies will help the successful execution of the project. This will be further supported by the work of the Safety, Regulatory and Ethics Board.

S4C governance includes coordination team and Boards, as patients advisory board (PAB) chaired by Eurordis, scientific advisory board, multistakeholder board (MSEB), ethical and legal and safety team (ELST), and other, as described in detail in Fig 3.

## Conclusions and perspectives

The clinical and genetic heterogeneity of RDs require many and diverse information for correctly identifying RDs patients so we believe that the impact of S4C technological developments and pipeline design will have a profound transformative effect in EU (and not only) on how we can utilize the data and pave the way for future endeavors in RD, as well as in genomic medicine in general [14].

gNBS pilot studies (TREAT and ACT) will involve the largest EU population never studied until now (about 25.000 infants) providing a unique dataset which will be stored in the S4C platform and open to be further shared in other EU contexts. The datasets generated by S4C include population genetic data (genotypes of genes included in TREAT and ACT panel), genome data from early symptomatic babies, phenotype data defined via AI and aggregate phenomics defined by the new developed algorithms. In addition, more focused datasets on muscle imaging or specific phenotypes derived from EHRs’ analysis will be collected. These unique datasets will grant the possibility to have a variety of information about different EU population, with the outstanding opportunity to correlate genotypes with phenotypes. S4C dataset will greatly improve the understanding about RD profiles in EU thus will be helping in designing a variety of health and therapy policies related to RDs, from population polymorphisms to very rare variants, and associated phenomics [15, 16]. Although it is difficult to predict the opt-in rate from participating expecting parents (and preference studies will also serve to address eventual criticisms) it is extremely unlikely that gNBS enrolment will be insufficient, based on regional birth rates and an estimated opt-in rate of 10% on overall births is more than achievable. Thus, we expect an opt-in rate of 85% on all enrolled expecting couples.

One of the major outputs expected from the gNBS studies is the identification of criteria gene/disease selection to be hopefully included into National NBS programs. The design of the TREAT and ACT panels will be based on a robust process based on several RD aspects, an also considering other similar initiatives protocols, as, for example, Genomics England [17], BeginNGS [18], and BabySeq [19]. The designing of the ACT panel will be informed by stakeholder consultations, such as the Rare Barometer EURORDIS survey program, which brings together close to 15,000 rare disease patients, family members and carers who share their experiences and opinions on the issues that matter to the rare disease community.

We are rapidly evolving toward a fully digitalized health system and comparing EHRs minimal contents among S4C clinical partners and different countries and addressing some bottlenecks as the modalities and privacy aspects of inclusion of genetic data into the digital systems, will enormously impact on the future European health data management with repercussion on national health systems. Our approach is harmonized with the EU health data space initiative [20] just launched. Collecting health data in a single data space is intuitively very important to allow aggregate data analysis and to ensure interoperability of the various datasets. Lastly, meta-symptom checker digital task will empower Health Care Providers (HCPs) and patients to facilitate assessing the appropriate pathway of diagnosis. To do that and to build the meta-symptom checker app, by horizon-scan of existing algorithms, we will assess the benefit of the Virtual Platform, again following the directives of the EU to improving health digitalization.

The possibility to design innovative gNBS is due to the fantastic work made and results obtained by the metabolic NBS initiatives now fully translated and embedded into the health systems. Thanks to the work done, NBS is now regarded as an integrated system, to gain equity, promote information, care, support, monitoring, and management, thus good practice NBS. This should be obviously applied to gNBS to prevent negative effects from outweighing the benefits and improve the outcome for patients and their families [21]. We like to underline that metabolic and gNBS should be considered distinct but integrated layers of screening. gNBS will not replace metabolic NBS but it is expected to increase the current offer by several orders of magnitude, ensuring the screening of thousand different RDs. Indeed, gNBS has the potentiality to investigate the entire genome, and consequently (and theoretically) all disease genes underlining RDs, therefore, able to include a broader spectrum of disorders, which is not detectable by metabolite or tandem mass spectrometry methods. It will remain substantial and important to define gNBS modalities, legal, ethical and privacy issues, and RD eligibility.

The tools developed by Screen4Care aim to stimulate digital transformation within the healthcare system and improve diagnostics for RDs. This IMI 2 JU research project strives to empower and inform patients and their families, to connect the rare disease communities across Europe and beyond, and to involve all stakeholders in the decision-making processes towards an accelerated proper diagnosis for PLWRD. The Sceen4Care consortium is driven by a will to improve equity and equality for the PLWRD which is an underserved community. Everyone deserves to be cared for, and care begins with diagnosis.

When successfully validated, the S4C gNBS and digital frameworks might be highly and flexibly translatable into the diverse European health systems.

## Abbreviations

AI: Artificial Intelligence
DBS: dried blood spots
EHR: electronic health records
ELST: Ethical, Legal and Safety Team
ERN: European Reference Network
EU: European Union
FAIR: Findable, Accessible, Interoperable, Reusable
GDPR: General Data Protection Regulation
gNBS: genetic newborn screening
HCP: Healthcare Provider
ML: Machine Learning
PAB: Patient Advisory Board
PLWRD: persons leaving with rare diseases
R&D: Research and Development
RD: Rare Disease
S4C: Screen4Care
WGS: whole genome sequencing
WP: Workpackage

## References

[1] Nguengang Wakap S, Lambert DM, Olry A, Rodwell C, Gueydan C, Lanneau V et al. Estimating cumulative point prevalence of rare diseases: analysis of the Orphanet database. Eur J Hum Genet. 2020;28:165–173. doi: 10.1038/s41431-019-0508-0 31527858 PMC6974615

[2] The Power of being counted in: Rare-X Website. https://rare-x.org/wp-content/uploads/2022/05/be-counted-052722-WEB.pdf, Accessed 29 December 2022.

[3] Haendel M, Vasilevsky N, Unni D, Bologa C, Harris N, Rehm H et al. How many rare diseases are there? Nat Rev Drug Discov. 2020;19:77–8. doi: 10.1038/d41573-019-00180-y 32020066 PMC7771654

[4] Kaufmann P, Pariser AR, Austin C. From scientific discovery to treatments for rare diseases—the view from the National Center for Advancing Translational Sciences—Office of Rare Diseases Research. Orphanet J Rare Dis. 2018;13:196. doi: 10.1186/s13023-018-0936-x 30400963 PMC6219030

[5] EURORDIS Website. www.eurordis.org, Accessed 29 December 2022.

[6] Rare Disease Facts in: Global genes Website. https://globalgenes.org/rare-disease-facts/, Accessed 29 December 2022.

[7] Bauskis A, Strange C, Molster C, Fisher C. The diagnostic odyssey: insights from parents of children living with an undiagnosed condition. Orphanet J Rare Dis. 2022;17:233. doi: 10.1186/s13023-022-02358-x 35717227 PMC9206122

[8] Yang G, Cintina I, Pariser A, Oehrlein E, Sullivan J, Kennedy A. The national economic burden of rare disease in the United States in 2019. Orphanet J Rare Dis. 2022;17:163. doi: 10.1186/s13023-022-02299-5 35414039 PMC9004040

[9] King JR, Notarangelo LD, Hammarström L. An appraisal of the Wilson & Jungner criteria in the context of genomic-based newborn screening for inborn errors of immunity. J Allergy Clin Immunol. 2021;147:428–438. 33551024 10.1016/j.jaci.2020.12.633 PMC8344044

[10] Andermann A, Blancquaert I, Beauchamp S, Déry V. Revisiting Wilson and Jungner in the genomic age: a review of screening criteria over the past 40 years. Bull World Health Organ. 2008;86:317–9. doi: 10.2471/blt.07.050112 18438522 PMC2647421

[11] International Joint Recommendations to address specific needs of Undiagnosed Rare Disease Patients, October2016. http://download2.eurordis.org.s3.amazonaws.com/documents/pdf/Undiagnosed-International-Joint-Recommendations.pdf, Accessed 29 December 2022.

[12] Bavisetty S, Grody WW, Yazdani S. Emergence of pediatric rare diseases: Review of present policies and opportunities for improvement. Rare Dis. 2013;1:e23579. doi: 10.4161/rdis.23579 25002987 PMC3932940

[13] Pomerantz A, Sahlin K, Vasiljevic N, Seah A, Lim M, Humble E et al. Rapid in situ identification of biological specimens via DNA amplicon sequencing using miniaturized laboratory equipment. Nat Protoc. 2022 Jun;17:1415–1443. doi: 10.1038/s41596-022-00682-x 35411044

[14] Toma M, Felisi M, Bonifazi D, Bonifazi F, Giannuzzi V, Reggiardo G et al. Paediatric Medicines in Europe: The Paediatric Regulation-Is It Time for Reform? Front Med (Lausanne). 2021;8:593281. doi: 10.3389/fmed.2021.593281 33604345 PMC7884470

[15] Committee for Orphan Medicinal Products and the European Medicines, Westermark K, Holm BB, Söderholm M, Llinares-Garcia J, Rivière F et al. European regulation on orphan medicinal products: 10 years of experience and future perspectives. Nat Rev Drug Discov. 2011;10:341–9. doi: 10.1038/nrd3445 21532564

[16] Report of Conference “Medicines for Rare Diseases and Children: Learning from the Past, Looking to the Future”, June 2019. https://health.ec.europa.eu/system/files/2019-07/ev_20190617_report_en_0.pd, Accessed 29 December 2022.

[17] Genomics England Website. https://www.genomicsengland.co.uk, Accessed 29 December 2022.

[18] Begin NGS Website. https://radygenomics.org/begin-ngs-newborn-sequencing, Accessed 29 December 2022.

[19] Ceyhan-Birsoy O, Murry JB, Machini K, Lebo MS, Yu TW, Fayer S, et al. Interpretation of Genomic Sequencing Results in Healthy and Ill Newborns: Results from the BabySeq Project. Am J Hum Genet. 2019 Jan 3;104(1):76–93. doi: 10.1016/j.ajhg.2018.11.016 30609409 PMC6323417

[20] Website of the European Commission. https://health.ec.europa.eu/ehealth-digital-health-and-care/european-health-data-space_en, Accessed 29 December 2022.

[21] Scarpa M, Bonham JR, Dionisi-Vici C, Prevot J, Pergent M, Meyts I, et al. Newborn screening as a fully integrated system to stimulate equity in neonatal screening in Europe. Lancet Reg Health Eur. 2022 Jan 28;13:100311. doi: 10.1016/j.lanepe.2022.100311 35199083 PMC8841274

