# Supplementary material for: Genetic newborn screening and digital technologies: A project protocol based on a dual approach to shorten the rare diseases diagnostic path in Europe
Source: PLoS One. 2023 Nov 22;18(11):e0293503. doi: 10.1371/journal.pone.0293503 (PMC10664952; doi:10.1371/journal.pone.0293503)
Supplement: Peer review history

# Editor report: Decision Letter 0

**DOI:** 10.1371/journal.pone.0293503.r001

**Authors:** Johanna Pruller

16 Oct 2023

Genetic newborn screening and digital technologies: a project protocol based on a dual approach to shorten the Rare Diseases diagnostic path in Europe

PONE-D-23-26393

Dear Dr. Ferlini,

We’re pleased to inform you that your manuscript has been judged scientifically suitable for publication and will be formally accepted for publication once it meets all outstanding technical requirements.

Within one week, you’ll receive an e-mail detailing the required amendments. When these have been addressed, you’ll receive a formal acceptance letter and your manuscript will be scheduled for publication.

An invoice for payment will follow shortly after the formal acceptance. To ensure an efficient process, please log into Editorial Manager at http://www.editorialmanager.com/pone/, click the 'Update My Information' link at the top of the page, and double check that your user information is up-to-date. If you have any billing related questions, please contact our Author Billing department directly at authorbilling@plos.org.

If your institution or institutions have a press office, please notify them about your upcoming paper to help maximize its impact. If they’ll be preparing press materials, please inform our press team as soon as possible -- no later than 48 hours after receiving the formal acceptance. Your manuscript will remain under strict press embargo until 2 pm Eastern Time on the date of publication. For more information, please contact onepress@plos.org.

Kind regards,

Johanna Pruller, Ph.D.

Associate Editor

PLOS ONE

Journal Requirements:

1. Thank you for stating the following financial disclosure: 

"S4C has received funding from the Innovative Medicines Initiative 2 Joint Undertaking (JU) under grant agreement No 101034427. The JU receives support from the European Union’s Horizon 2020 research and innovation programme and EFPIA."

Please state what role the funders took in the study.  If the funders had no role, please state: "The funders had no role in study design, data collection and analysis, decision to publish, or preparation of the manuscript." 

If this statement is not correct you must amend it as needed. 

Please respond by return e-mail so that we can amend your financial disclosure and competing interests on your behalf.

# Editor report: Acceptance letter

**DOI:** 10.1371/journal.pone.0293503.r002

**Authors:** Johanna Pruller

25 Oct 2023

PONE-D-23-26393 

Genetic newborn screening and digital technologies: a project protocol based on a dual approach to shorten the Rare Diseases diagnostic path in Europe 

Dear Dr. Ferlini:

I'm pleased to inform you that your manuscript has been deemed suitable for publication in PLOS ONE. Congratulations! Your manuscript is now with our production department. 

If your institution or institutions have a press office, please let them know about your upcoming paper now to help maximize its impact. If they'll be preparing press materials, please inform our press team within the next 48 hours. Your manuscript will remain under strict press embargo until 2 pm Eastern Time on the date of publication. For more information please contact onepress@plos.org.

If we can help with anything else, please email us at plosone@plos.org. 

Thank you for submitting your work to PLOS ONE and supporting open access. 

Kind regards, 

PLOS ONE Editorial Office Staff

on behalf of

Dr. Johanna Pruller 

Staff Editor

PLOS ONE
